# Accuracy of ancestral state reconstruction for non-neutral traits

**DOI:** 10.1038/s41598-020-64647-4

**Published:** 2020-05-06

**Authors:** Barbara R. Holland, Saan Ketelaar-Jones, Aidan R. O’Mara, Michael D. Woodhams, Gregory J. Jordan

**Affiliations:** 1 School of Natural Sciences, University of Tasmania, Private Bag 55, Hobart, Tas 7001 Australia; 2 School of Health Sciences, University of Tasmania, Private Bag 121, Hobart, Tas 7001 Australia

**Keywords:** Evolution, Phylogenetics

## Abstract

The assumptions underpinning ancestral state reconstruction are violated in many evolutionary systems, especially for traits under directional selection. However, the accuracy of ancestral state reconstruction for non-neutral traits is poorly understood. To investigate the accuracy of ancestral state reconstruction methods, trees and binary characters were simulated under the BiSSE (Binary State Speciation and Extinction) model using a wide range of character-state-dependent rates of speciation, extinction and character-state transition. We used maximum parsimony (MP), BiSSE and two-state Markov (Mk2) models to reconstruct ancestral states. Under each method, error rates increased with node depth, true number of state transitions, and rates of state transition and extinction; exceeding 30% for the deepest 10% of nodes and highest rates of extinction and character-state transition. Where rates of character-state transition were asymmetrical, error rates were greater when the rate away from the ancestral state was largest. Preferential extinction of species with the ancestral character state also led to higher error rates. BiSSE outperformed Mk2 in all scenarios where either speciation or extinction was state dependent and outperformed MP under most conditions. MP outperformed Mk2 in most scenarios except when the rates of character-state transition and/or extinction were highly asymmetrical and the ancestral state was unfavoured.

## Introduction

Ancestral state reconstruction is a fundamental tool for exploring evolution because it provides estimates of otherwise unobservable processes^1^. Given a character – any heritable trait for which there is a reasonable hypothesis that all states are homologous – ancestral state reconstruction estimates character states for both recent and deep time ancestors. Ancestral state reconstruction methods combine information about evolutionary relationships from a phylogenetic tree with the observed state of a character for each tip (each terminal node, often an extant species). Many ancestral state reconstruction methods have been developed, including parsimony-based methods, which minimise the total number of changes of state across the tree, and a range of likelihood-based approaches that use stochastic Markov models of character change. Generally, ancestral state reconstruction is performed after a phylogenetic tree has been constructed, the one main exception to this being Bayesian phylogeography methods that attempt to co-estimate the tree and the ancestral geographical state^2^. Almost all methods for ancestral state reconstruction assume that the given tree is congruent with the phylogeny on which the character evolved^3,4^, and ancestral state reconstruction is known to be biased in cases where this is not true^5^.

Most methods of ancestral state reconstruction assume that the character under consideration is neutral, and that the evolutionary process has not changed across the phylogenetic tree. Although these assumptions make data analysis more straight-forward, they can impact on the accuracy and biological validity of findings^6–8^. Known sources of bias in both phylogenetic tree construction and ancestral state reconstruction include changes in rates and processes of evolution across the tree^1,9–12^, dependence between the state of a character and the likelihood of lineage extinction or speciation^13,14^, evolutionary convergence^15–17^ and hemiplasy^18^. In the specific case of ancestral sequence reconstruction, recombination^19^ and substitution-model misspecification^20^ are also known to bias results.

The assumption of neutrality has the important consequence that the estimate of the phylogenetic tree is independent of the estimate of ancestral states. However, ancestral state reconstruction is often applied to functionally important traits, which are unlikely to be neutral. Such traits are likely to be susceptible to natural selection, and the state of the trait may influence the probability of extinction or speciation. Systems that have undergone directional evolution – a non-random shift in the distribution of traits over time^21^ – and/or systematic extinction – in which species with one state go extinct at a greater rate than species with other states – are particularly problematic because they may create systematic biases. These potential biases are likely to be important for traits influenced by substantial and widespread temporal changes in environmental variables such as atmospheric CO_2_^22^, temperature^23^, predominant vegetation type^24,25^, or aridity. Similarly, radiations of major groups of organisms (e.g. multicellular life, terrestrial vertebrates, mammals, angiosperms, as well as more local radiations) almost certainly induced major changes in the selective regimes for other groups of organisms present at those times. Numerous traits have been proposed to influence diversification^26,27^, this also has potential to bias ancestral state reconstruction.

The *SSE (*State Speciation Extinction) models for ancestral state reconstruction aim to overcome some of these problems by allowing rates of character-state transition, extinction and speciation to depend on the current state of a character^28^. The original BiSSE model^29^ dealt with a single binary trait that influenced speciation and extinction rates. The MuSSE and QuaSSE models^30^ provided extensions to multistate and quantitative characters respectively. There is some evidence that BiSSE provides better estimates of evolutionary parameters than models where character evolution occurs independently of the branching process^31^. However, the use of BiSSE models to test hypotheses of state-dependent evolution has been found to be prone to Type I error in cases where the trait considered does not affect speciation or extinction but other traits do^32^. To cope with this situation, the recently developed HiSSE model^33^ allows for diversification and extinction rates to depend on an unobserved state that may be correlated with the observed trait.

The effectiveness of BiSSE and the other *SSE models has been largely investigated with respect to inferring diversification dynamics^31^; their effectiveness for ancestral state reconstruction remains largely untested. There are some notable exceptions, the performance of BiSSE for ancestral state reconstruction in the context of testing for characters that evolve irreversibly has been examined^34^ and re-examinations of the BiSSE model in the context of reconstructing changes in parity mode in lizards found that ancestral state reconstruction was sensitive to model choice, rate heterogeneity, and the estimate of the underlying phylogenetic tree^12,35^. It is important to note that, inferring diversification dynamics (the most common use of *SSE models) cannot be accurate if ancestral state reconstruction is inaccurate.

In this paper, we conduct a simulation study to assess the accuracy of common methods of ancestral state reconstruction. We exploit the capacity of BiSSE to evolve characters and phylogenetic trees simultaneously, with state-dependent rates of character transition, speciation and extinction. In particular, by forcing the character state at the root of the tree to have a particular value, we can use BiSSE to generate characters that have undergone directional (i.e. non-stationary) evolution as well as systematic extinction. Because the true state of a character at each node in the tree is recorded in the trees generated by BiSSE, such trees provide an opportunity to test the accuracy of ancestral state reconstruction methods under far more realistic evolutionary scenarios than has been previously possible.

We therefore test scenarios with wide ranges of character-state conditional rates of speciation and extinction, and rates of character-state transition. The scenarios include ones in which rates of speciation, extinction, and character-state transition, range from equal to highly asymmetrical, but the rates are all empirically realistic and within theoretical limits on recoverability^36^. We assess both overall accuracy and how accuracy depends on node depth for three different approaches for inferring ancestral states: parsimony, likelihood under the Mk2 model^37^ (henceforward Mk2), and likelihood under BiSSE^29^. Specifically, we address the questions: (1) How much does accuracy of ancestral state reconstruction decrease with increasing rates of character-state transition and increasing node depth? (2) Does accuracy decrease with increasing rates of extinction? (3) Is accuracy lower when the ancestral state is unfavoured compared to otherwise comparable scenarios in which the ancestral state is favoured? This hypothesis is addressed by assessing the impacts of preferential extinction of species carrying the ancestral state (selective extinction), preferential transition from the ancestral to the derived state (directional evolution) and preferential speciation of the derived state. (4) Does BiSSE outperform maximum parsimony and Mk2 under conditions of asymmetrical state-dependent rates of speciation, extinction and character-state transition?

## Methods

### Simulation parameters

Simulated phylogenetic trees were created using the Binary State Speciation and Extinction (BiSSE) model^29^, in which a tree and a binary character are evolved simultaneously. The initial state at the root of all trees was constrained to be 0 (so 0 is the ancestral state and 1 the derived state). The rates of speciation (*λ*_0_ and *λ*_1_) and rates of extinction (*µ*_0_ and *µ*_1_) are each defined according to the state of the character, and the character changes state between states 0 and 1 at rates *q*_01_ (forward) and *q*_10_ (reverse).

We created trees with 400 tips for scenarios that included both symmetric and asymmetric rates of extinction and character-state transition. We note that Davis *et al*.^31^ found that more than 300 tips were required for accurate parameter inference with BiSSE models. 500 trees were created for each scenario. Extinction rates (*µ*_0_ and *µ*_1_) were taken from in the set {0.01, 0.25, 0.5, 0.8} (4 × 4 = 16 situations); character-state transition parameters (*q*_01_ and *q*_10_) from {0.01, 0.05, 0.1} (3 × 3 = 9 situations); and speciation rates covered five pairs of values {(λ_0_ = 0.2; λ_1_ = 1.8),(λ_0_ = 0.5; λ_1_ = 1.5), (λ_0_ = 1; λ_1_ = 1), (λ_0_ = 1.5; λ_1_ = 0.5), (λ_0_ = 1.8; λ_1_ = 0.2)} (5 situations). This gave a total of 720 (16 × 9 × 5) different scenarios.

We constrained the average rate of speciation, (*λ*_0_ + *λ*_1_)/2, to be one. Rates of character-state transition and extinction were then chosen to be biologically reasonable relative to speciation. Rates of character-state transition were chosen so that they were high enough to produce non-constant characters in almost all trees, yet not so high that ancestral state reconstruction was impossible^7^. Thus, our modelling restricted $\frac{\lambda }{q}$ to values of at least 10 because the accuracy of ancestral state reconstruction at the root is theoretically expected to drop off steeply when ratios of character-state transition to speciation rate $(\frac{\lambda }{q})$ are less than 6 for parsimony methods or less than 4 for likelihood models^36^. Previous work has found evidence that in some groups of organisms the rate of extinction relative to speciation is close to one^38,39^, but we set our maximum extinction rate to 0.8; it is difficult to simulate trees for higher extinction rates as a large proportion of cases go extinct rather than reach the required number of tips.

Simulations were carried out using the function *tree.bisse* in the package *diversitree*^40^ in R^41^. Our computations made use of the Gnu *parallel* tool^42^. For each scenario we repeated the simulations until we had 500 trees, i.e. we conditioned on the process not going extinct. Overall, we conducted an average of 2565 extra simulations for each scenario to get 500 trees. Of the 720 simulation scenarios, 360 have the property that extinction rates *µ*_0_ and *µ*_1_ are strictly smaller than the corresponding state-dependent speciation rates λ_0_ and λ_1_, so that clades were expected to be constantly expanding. For expanding clade scenarios, the average number of extra simulations required was 335 and for non-expanding clade scenarios the average was 4794.

For the purposes of simplifying the presentation of results and studying the effect of tree size, we define a set of “corner case” parameter settings, having *q*_01_ and *q*_10_ in {0.01, 0.1}, *µ*_0_ and *µ*_1_ in {0.01, 0.8} and *µ*_i_ ≤ λ_i_. This gives 32 parameter settings. For these corner cases, we in addition analysed 100 and 1600 tip trees (using only 200 trees per parameter set for 1600 tip trees, to save computation time).

Any trees in which the character was invariant at the tips were excluded from further analysis. Although, it is certainly possible to make mistaken inferences about ancestral states in these cases, they are not typically considered interesting enough to do ancestral state reconstruction on, so we chose to exclude them. On average 13 of the 500 generated trees were excluded for this reason (for the 360 expanding clade scenarios the average number of trees excluded was 5.3). The worst such parameter setting for expanding clade scenarios excluded 16% of trees (for λ_0_ = 1.8; λ_1_ = 0.2; *µ*_0_ = 0.01; *µ*_1_ = 0.01; *q*_01_ = 0.01; *q*_10_ = 0.1, i.e. with both speciation and mutation strongly biased to state 0).

### Methods of ancestral state reconstruction

For each tree we applied three methods of ancestral state reconstruction on the simulated character:

1. Maximum parsimony (MP) using the MPR function from the *ape* package^43^. This uses the method of Hanazawa *et al*.^44^ as modified by Narushima and Hanazawa^45^.
2. Likelihood under Mk2^37^ using the functions *make.mk2*, *find.mle* (method = “subplex”) and *asr.marginal* from the *diversitree* package^40^. This method allows *q*_01_ and *q*_10_ to differ, but does not allow for state-dependent extinction or speciation. To protect against the *find.mle* function getting trapped in a local optimum we performed optimization from three different random starting conditions. For each random start, the parameter estimates for *q*_01_ and *q*_10_ were initialised to their true values multiplied by a factor between 0.5 and 1.5.
3. Likelihood under BiSSE using the functions *make.bisse*, *find.mle* (method = “subplex”) and *asr.marginal* from the *diversitree* package^40^. The parameter estimates for *λ*_0_, *λ*_1_, *μ*_0_, *μ*_1_, *q*_01_ and *q*_10_ were initialised to three different random starting values using the same scheme as described above and subsequently optimized.

Marginal reconstructions of ancestral states (as carried out by *asr.marginal* for all our simulations) focus on one node at a time, they assign probabilities of a node being in a particular state by looking at the relative likelihood of the data when the node is fixed as state 0 versus 1. This is distinct from joint ancestral state reconstruction methods which attempt to reconstruct a complete history of transitions.

### Assessing the error rates of methods

We compared the true state (as recorded in the evolution of the simulated tree) with the state inferred by ancestral state reconstruction for all internal nodes in each tree. For binary characters, Mk2 and BiSSE yield a probability of the node having the state 1, whereas parsimony records a direct estimate of state (0, 1 or ambiguous) for each node. To compare the different methods of ancestral state reconstruction we used two metrics of error rates. In the first, we converted Mk2 and BiSSE probabilities for each node into 0, 1, or ambiguous with this rule: if the probability was greater than 0.7, then the node state was assigned as 1, if the probability was less than 0.3, the state was assigned as 0, probabilities between 0.3 and 0.7 were considered as ambiguous. We then only considered “outright” errors, i.e. if the estimated and true states did not match. We refer to this as the *quantised score*. The second approach directly employed the node probabilities for Mk and BiSSE, and error scores of 0 (estimated and true values matched), 0.5 (ambiguous estimate) and 1 (estimated and true values were different) for MP. For example, if a node was estimated to have a 0.15 probability of being in state 0 and the simulated value was truly 0 then this would count as an error of 0.15. We refer to this as the *raw score*. For both approaches, the analyses employed means of the error scores for quantiles of node depth per tree, or for parameter setting scenarios.

For the simulations on 400 tip trees, we used logistic regression models – implemented using the *glm* function in R^41^ – to assess the impact of parameters on the probability of errors in ancestral state reconstruction for individual trees. The glms had continuous fixed effects for *µ*_0_, *µ*_1_, *q*_01_, *q*_10_, and the (natural) log of the true number of transitions in the tree, and categorical fixed effects for method (BiSSE, Mk2 or MP) and λ_0_. λ_0_ was treated as categorical as the effect was not linear. λ_1_ was not included in the model because its value was determined by the value of λ_0_. The response variable was the presence/absence of errors in the tree (i.e. the outright errors under the quantised scoring method). We fit models of the form

$$\begin{array}{l}{\rm{Error}} \sim ({\mu }_{0}+{\mu }_{1}+{q}_{01}+{q}_{10}+log({\rm{transitions}}))\ast {\rm{Method}},\,({\rm{Model}}\,1)\\ {\rm{Error}} \sim ({\mu }_{0}+{\mu }_{1}+{q}_{01}+{q}_{10}+{\lambda }_{0}+log({\rm{transitions}}))\ast {\rm{Method}}\,({\rm{Model}}\,2)\end{array}$$

There were models with more interaction terms that gave better AIC scores, but as our prime concern was understanding interactions between methods and parameters we preferred to analyse these relatively simpler models.

## Results

### Reconstructions for moderate sized trees (400 tips)

**Figure 1:**
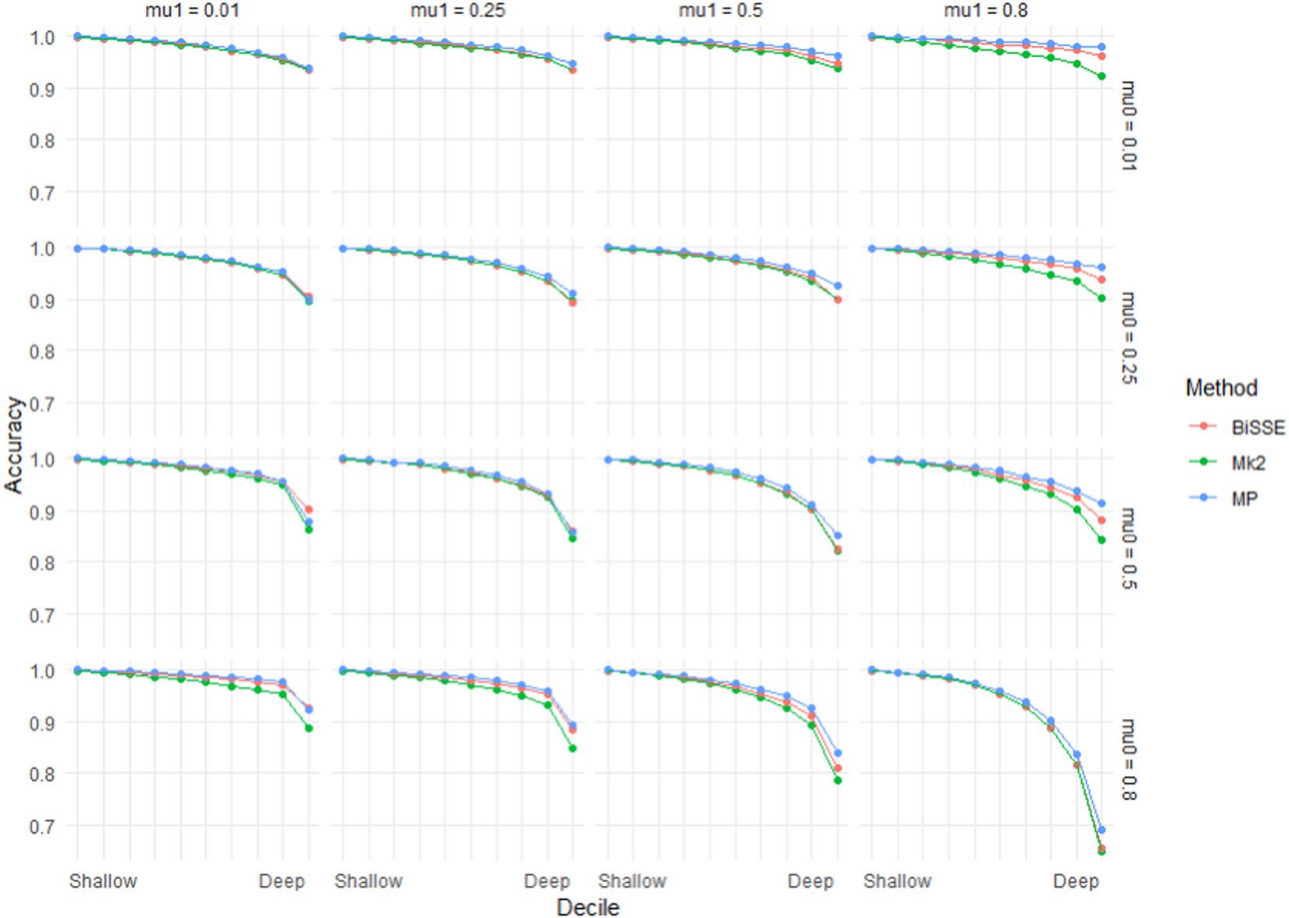
Accuracy (based on raw scores) for scenarios with equal rates of speciation (λ_0_ = 1) and high but symmetric rates of character transition (q_01_ = q_10_ = 0.1). Note that scenarios below the diagonal (running top-left to bottom-right) have higher errors rates than those above the diagonal – these are scenarios in which the ancestral state is more likely to go extinct.

**Figure 2:**
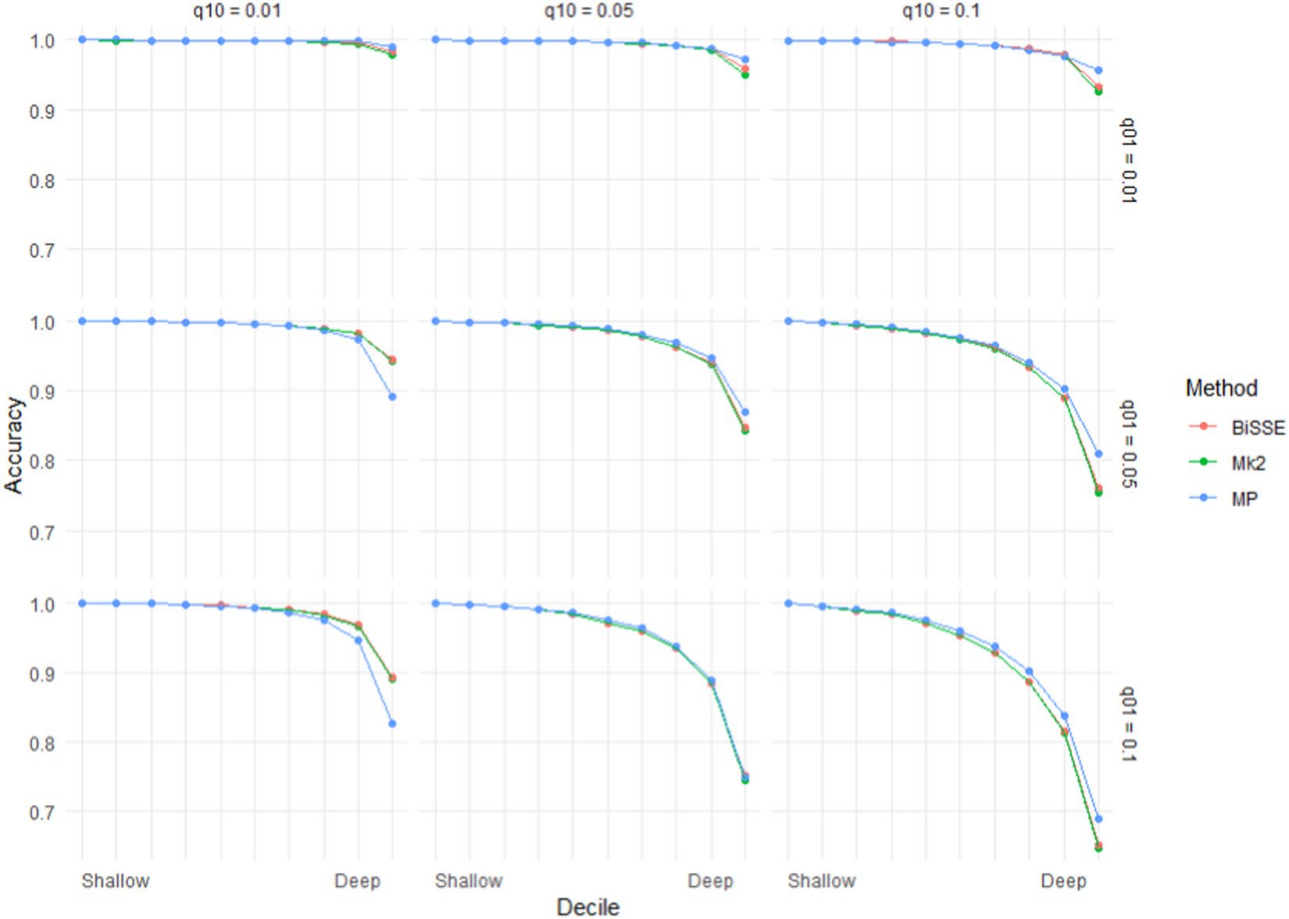
Accuracy (based on the raw scores) for scenarios with equal rates of speciation (λ_0_ = 1) and high but symmetric rates of extinction (µ_0_ = µ_1_ = 0.8). Note that scenarios below the diagonal (running top-left to bottom-right) have higher errors rates than those above the diagonal – these are scenarios in which transitions from the ancestral state are more frequent than transitions to the ancestral state.

For each of the MP, Mk2 and BiSSE reconstruction methods, ancestral state reconstruction performed worst for deep nodes in trees evolved under the highest rate of extinction of species with the ancestral state (*µ*_0_ = 0.8), and the highest rate of character-state transition from the ancestral to derived state (i.e. q_01_ = 0.1) (Figs. 1 and 2). Error rates of shallow nodes (i.e. those near the tips) were very low under all scenarios, but the basal 10% of nodes for the worst scenarios had mean error rates over 30% (Figs. 1 and 2).

As seen in Figs. 1 and 2, error rates showed asymmetry depending on whether the root node was in the unfavoured or favoured state. Thus, error rates were higher when µ_0_ > µ_1_ than when µ_1_ > µ_0_: i.e. accuracy was more affected by extinction of species with the root state than extinction of species with the derived state. Similarly, error rates when q_01_ > q_10_ were greater than under comparable scenarios when q_10_ > q_01_, which implies that evolution favouring transitions to the derived state has a greater impact on accuracy than reversions to the root state. Error rates for very deep nodes (e.g. the basal 1% of nodes) were much greater again, roughly twice as great as those for the basal 10% of nodes for the same scenario (Supplementary Materials Fig. S1).

**Figure 3:**
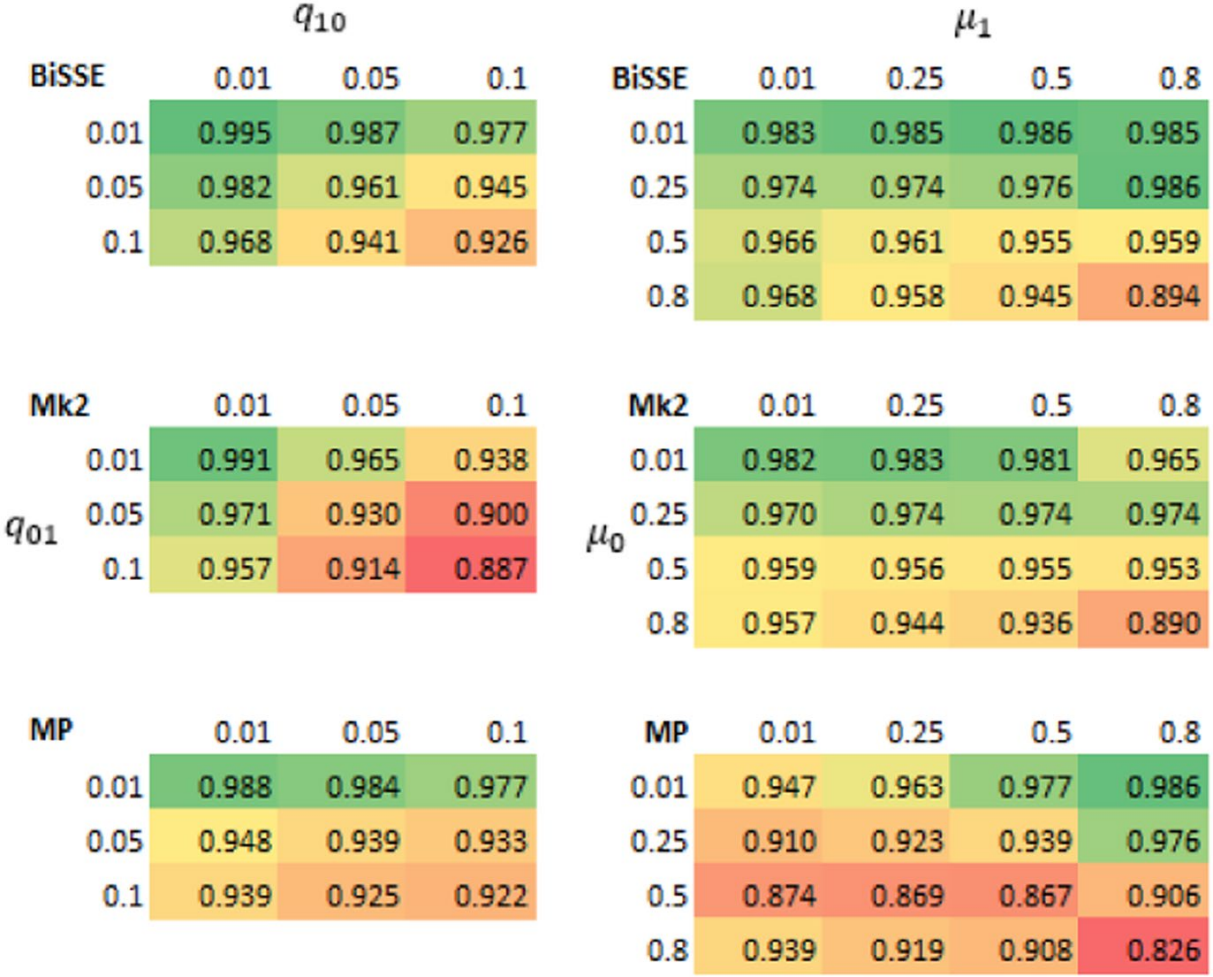
Accuracy (based on raw scores) for the deepest decile, for (left panel) scenarios with λ_0_ = λ_1_ = 1, µ_0_ = 0.8 and µ_1_ = 0.01 and (right panel) scenarios with λ_0_ = λ_1_ = 1, q_01_ = 0.1 and q_10_ = 0.01. The colour scale represents accuracy with greener shades representing higher accuracy, it is normalised separately for the left and right panels. Errors rates for MP are noticeably more affected by the q_01_ and µ_0_ transition rates than the q_10_ and µ_1_ transition rates.

For MP in particular accuracy at the deepest nodes was reduced when the ancestral state was unfavoured due to both extinction and state-change asymmetry (Fig. 3). This makes sense as MP implicitly assumes that state change is symmetrical.

**Figure 4:**
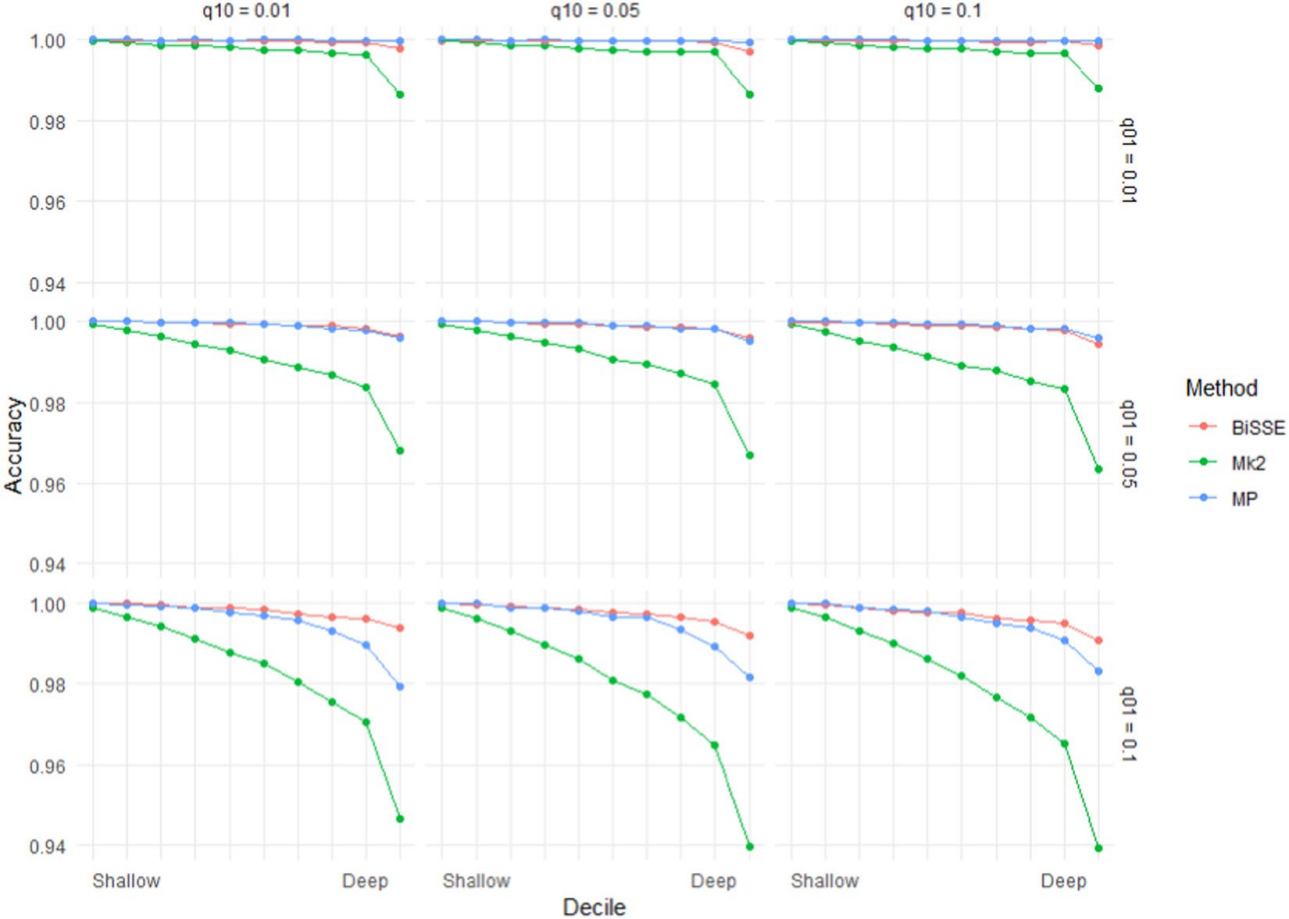
Mean accuracy (based on raw scores) for scenarios with unequal rates of speciation (λ_0_ = 1.8, λ_1_ = 0.2) and low extinction rates (${\mu }_{0}$ = ${\mu }_{1}$ = 0.01). Increasing q_01_ has a greater impact on accuracy than increasing q_10_.

Asymmetry in speciation rates had a bigger effect on the accuracy of Mk2 than MP or BiSSE (Fig. 4). BiSSE was usually the most accurate method in scenarios with asymmetric speciation rates; there were scenarios (q_01_ = 0.01) where MP was slightly more accurate, but in these cases both methods were close to 100% accurate. Rates of speciation had an unintuitive impact – many scenarios with asymmetrical rates of speciation (i.e. λ_0_ ≠ λ_1_) showed lower error rates than related scenarios in which λ_0_ = λ_1_. However, this appears to be linked to very unequal numbers of states 0 and 1 in the tips under scenarios with asymmetrical speciation: error rates were significantly higher when states 0 and 1 were more-or-less similarly represented among the tips than when one state dominated (Supplementary Materials Fig. S2).

**Figure 5:**
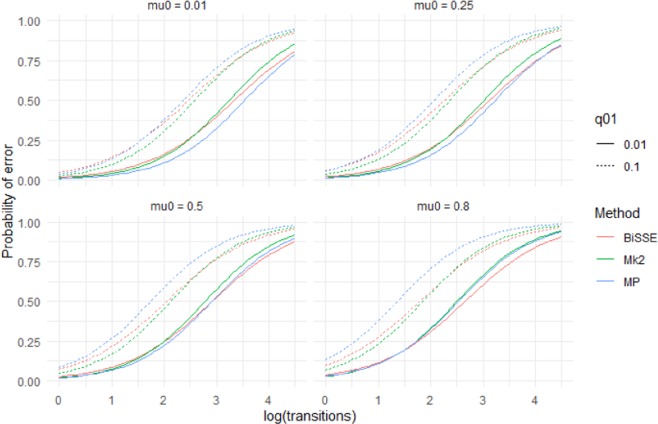
Predictions from a logistic regression model (Model 1) that fit the presence of an incorrect inferred state to the categorical main effect of method, the continuous main effects of log(#transitions), µ_0_, µ_1_, q_01_, q_10_ and interaction effects between Method and the other variables. The model was fit to data from 400 tip simulations across 500 repetitions of the 144 scenarios where λ_0_ = λ_1_ = 1. For predictions ${\mu }_{1}$= 0.25 and q_10_ = 0.05. Different panels show predictions for different values of µ_0._.

The results based on raw scores were supported by logistic regression models based on presence/absence of misidentified nodes in individual trees, i.e. outright errors in the quantised scores (Supplementary Materials S1)). For model (1), all main effects were significant (*P* < 0.001) and all interaction terms were significant (P < 0.05). The effect of *µ*_0_ exceeded that of *µ*_1_. MP’s predicted probability of making an ancestral state reconstruction error is more affected by q_01_ and ${\mu }_{0}$ than Mk2 or BiSSE (Fig. 5, Supplementary Materials Fig. S3). Mk2’s predicted performance deteriorates fastest with increasing number of transitions (Supplementary Materials Table S1). Figure 5 shows that different methods are predicted to perform best, in the sense of producing trees with no outright errors, for different scenarios. When q_01_ and ${\mu }_{0}$ are both low MP does well. For q_01_ = 0.1 Mk2 has the lowest probability of errors except for scenarios with many transitions. In the case where q_01_ and ${\mu }_{0}$ are both high BiSSE performs best.

**Figure 6:**
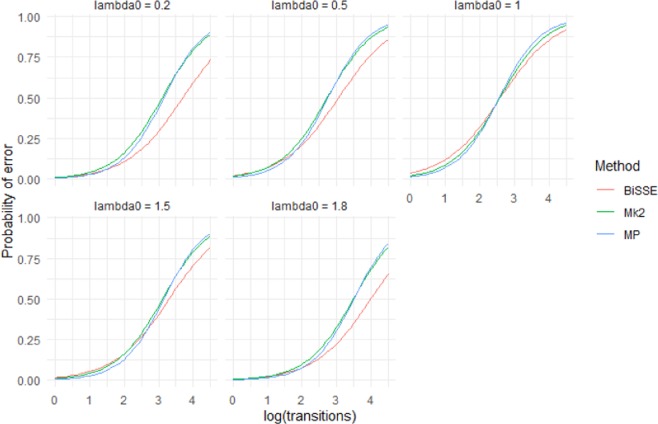
Predictions from a logistic regression model (Model 2) that fit the presence of an incorrect inferred state to the categorical main effects of method and λ_0_, the continuous main effects of log(#transitions), µ_0_, µ_1_, q_01_, q_10_ and interaction effects between Method and the other variables. The model was fit to data from 400 tip simulations across 500 repetitions of all 720 scenarios. For predictions *µ*_0_ = ${\mu }_{1}$= 0.25 and *q*_01_ = q_10_ = 0.05. Different panels show predictions for different values of λ_0._ Note that λ_0_ = 1 is the symmetrical scenario where λ_0_ = λ_1_ (shown in greater detail in Fig. 5), in the asymmetrical scenarios BiSSE outperforms Mk2 and MP.

The performance of the three methods (BiSSE, Mk2 and MP) did not vary greatly for low numbers of transitions (Fig. 6). However, BiSSE performed better than the other two methods at high numbers of transitions particularly when rates of speciation were asymmetrical (Fig. 6).

For analyses with the quantised scores, MP estimates had more ambiguous nodes than BiSSE or Mk2 (Supplementary Materials Fig. S4, perhaps because relatively arbitrary thresholds (i.e. 0.3 < P < 0.7) were used to determine which nodes were considered ambiguous for BiSSE and Mk2. The quantised error rates for BiSSE and Mk2 may well be smaller than those of MP under a more relaxed threshold for identifying ambiguous nodes.

### Effect of tree size

Trees with 100 tips had significantly higher error rates (based on mean raw score per “corner case” scenario) than trees with 400 or 1600 tips (Supplementary Materials Fig. S5(A)), but there was no significant difference in the performance ratio among methods. There was little difference in percentage error rates for trees with 1600 tips compared to trees with 400 tips (Supplementary Materials Fig. S5(B)). Mean depth of the simulated trees increased with number of tips (7.18, 9.22 and 11.09 units for 100, 400 and 1600 tips, respectively).

## Discussion

This work has clear implications for ancestral state reconstruction of labile characters and characters under directional selection. It also has implications for *SSE methods that rely on accurate ancestral state reconstruction to infer diversification dynamics. Although maximum likelihood (Mk2), maximum parsimony and BiSSE-based ancestral state reconstruction were reliable for both shallow nodes and deep nodes under scenarios with few transitions and extinctions (Figs. 1 and 2), under high rates of character-state transition and/or extinction error rates were high for deep nodes, especially the deepest nodes (Supplementary Materials Fig. S1). For some scenarios, error rates approached 50%. Also, characters with high levels of evolutionary lability were difficult to reconstruct – error rates increased strongly with the true number of transitions (Fig. 5). These high error rates are important because the highest state transition rates used here (*q* = 0.1) were considerably lower relative to speciation rate than thresholds identified as posing problems for ancestral state reconstruction using maximum parsimony and maximum likelihood^36^. The high error rates observed for both the more basal nodes of the trees and for trees with relatively high numbers of transitions are important because many ancestral state reconstruction studies identify multiple transitions, including transitions in basal parts of trees (Supplementary Materials Fig. S6).

These simulations indicate that directional evolution and systematic extinction have substantial impacts on ancestral state reconstruction, and significant errors may be expected in reconstructing traits that have been influenced by substantial and widespread changes in environment. The greater influence of µ_0_ and q_01_ than µ_1_ and q_10_ on the average accuracy (Figs. 1–4) and on the presence of errors (Figs. 5 and 6, Supplementary Materials Table S1) indicate that error rates were higher when there was preferential extinction of species with the ancestral state and/or preferential evolution towards the derived state over reversion to the ancestral state. It makes sense that higher rates of reversions (q_10_) have less effect on accuracy, as such changes will generally occur at shallower node depth (because the root state has to change to the derived state before reversions can occur) and shallower nodes tend to be easier to accurately infer. Similarly, higher rates of extinction for the derived state will preferentially remove younger clades, and this younger part of the tree should be easier to infer even with reduced sampling. Our results are consistent with studies of experimentally evolved viruses showing that directional selection can cause biased ancestral state reconstruction of continuous characters under both maximum parsimony and maximum likelihood^46^.

In terms of raw error rates, BiSSE outperformed Mk2 across all 400 tip scenarios (although the methods performed similarly except in cases where speciation was state-dependent). BiSSE had lower error rates than MP except for scenarios with low numbers of transitions when overall error rates were very low for all methods. MP does well across many of the scenarios we tested with the exception of scenarios where the rate from the ancestral to derived state, *q*_01_, is high relative to *q*_10_ (Figs. 2 and 3). The presence of state-dependent speciation particularly effected the accuracy of Mk compared to MP and BiSSE. These results are congruent with Goldberg and Igic^34^, who found that BiSSE tended to perform better at identifying irreversible trait evolution (the extreme case of state-dependent rates of character transition) than Mk2. Goldberg and Igic^34^ did not directly test errors in ancestral state reconstruction, also they only used simulations with symmetrical rates of state dependent speciation and extinction, and quite low rates of extinction.

The results of our simulation study quantify the error rates of common ancestral state reconstruction methods under the range of biologically reasonable scenarios. These error rates were uniformly low for internal nodes near the tips (<1% across all scenarios for the two shallowest deciles). They were also low for nodes of intermediate depth (<2% across all scenarios for all but the deepest two deciles) when state transition and extinction rates were all low to moderate ($q\,\le 0.05$ and $\mu \le 0.25$, noting that this is relative to fixed speciation rates of 1). However, for the deep nodes in the tree, error rates were moderate to high for all methods, especially when rates of extinction and/or state transition were high. For scenarios with $q=0.1$ and $\mu \ge 0.5$ the mean error rates in the deepest 10% of nodes were 13% for Mk2 and ~5% for BiSSE and MP. For the most challenging scenario ($\lambda =1,\,\mu =0.8,\,q=0.1$) mean error rates in the deepest 10% of nodes were over 30% for all methods. Of the 720 simulation scenarios, and considering only the deepest decile, there were 62 scenarios where Mk2 had accuracy <90%, 35 scenarios where MP had accuracy <90% and 17 scenarios where BiSSE had accuracy <90%.

For some evolutionary scenarios outside the range of conditions covered in this study, the error rates may be even greater. Very high rates of character-state transition relative to speciation are known to cause failure of ancestral state reconstruction^36^. In addition, our analyses only employed time homogeneous rates of transitions or extinctions. However, changes in selective regimes are likely to induce changes in rates of state transition, and rates of speciation and/or extinction in real systems vary greatly among clades, with for example, the single species *Amborella trichopoda* likely to be sister to all other angiosperms (~400,000 species)^47^. The impacts of such changes on ancestral state reconstruction are unknown but are likely to be bad unless rate heterogeneity is explicitly accounted for^12^. Although methods implemented in corHMM^48^ allow for variation in rates of character-state transition, no methods allow for variable rates of extinction. Our results hint at the possible consequences of one important scenario – clades in which extinction substantially exceeds speciation for some period. Since error rates increase with extinction rate, error rates may be very high when extinction exceeds speciation. Thus, current methods of ancestral state reconstruction may be poor predictors for deep nodes in many real world evolutionary systems, and past major environmental changes should be carefully considered when interpreting ancestral state reconstruction of functionally important traits.

## Supplementary information

Supplementary Information.

Supplementary Materials .

## Data Availability

All data summary files generated or analysed during this study along with R code to reproduce the figures are included in the Supplementary Information files. Code for generating the raw data and producing summary files is available from https://github.com/MichaelWoodhams/bisse.
