# Supplementary material for: Accuracy of ancestral state reconstruction for non-neutral traits
Source: Sci Rep. 2020 May 6;10:7644. doi: 10.1038/s41598-020-64647-4 (PMC7203120; doi:10.1038/s41598-020-64647-4)
Supplement: Supplementary file 2 — Supplementary Materials . [file 41598_2020_64647_MOESM2_ESM.docx]

**Accuracy of ancestral state reconstruction for non-neutral traits**

**Barbara R. Holland*^1^,Saan Ketelaar-Jones^1^, Aidan R. O’Mara^2^, Michael D. Woodhams^1^, and Gregory J. Jordan^1^**

^1^School of Natural Sciences, University of Tasmania, Private Bag 55, Hobart, Tas. 7001, Australia.

^2^School of Health Sciences, University of Tasmania, Private Bag 121, Hobart, Tas. 7001, Australia

^*^ [Barbara.Holland@utas.edu.au](mailto:greg.jordan@utas.edu.au)

**Supplementary Materials**

**Table S1.** Summary of output for binary logistic regression model of the form

Presence of error ~ (q_01_ + q_10_ + mu_0_ + mu_1_ + log(transitions))* Method

Coefficient estimates are given in the link scale, i.e. they represent the effect on a log(odds) scale. Positive coefficients correspond to terms with a multiplicative effect on the odds of an error that is greater than 1. BiSSE was set as the baseline level of the Method factor.

| **Term** | **Estimate** | **Std. error** | **Z value** | **P-value** |
| --- | --- | --- | --- | --- |
| Intercept | -4.92213 | 0.04288 | -114.776 | < 0.001 |
| q_01_ | 11.83025 | 0.36219 | 32.663 | < 0.001 |
| q_10_ | 12.98848 | 0.29152 | 44.554 | < 0.001 |
| $\mu_{0}$ | 1.05633 | 0.03302 | 31.993 | < 0.001 |
| $\mu_{1}$ | 0.18429 | 0.03224 | 5.715 | < 0.001 |
| log(transitions) | 1.23054 | 0.01648 | 74.677 | < 0.001 |
| Method Mk2 | -0.34506 | 0.06236 | -5.533 | < 0.001 |
| Method MP | -0.68347 | 0.06435 | -10.622 | < 0.001 |
| q_01_:Method Mk2 | -1.61271 | 0.51806 | -3.113 | 0.002 |
| q_01_:Method MP | 5.97731 | 0.52522 | 11.381 | < 0.001 |
| q_10_:Method Mk2 | -0.94151 | 0.41681 | -2.259 | 0.024 |
| q_10_:Method MP | -1.32839 | 0.42732 | -3.109 | 0.002 |
| $\mu_{0}$:Method Mk2 | 0.25271 | 0.04734 | 5.339 | < 0.001 |
| $\mu_{0}$:Method MP | 0.6886 | 0.04857 | 14.177 | < 0.001 |
| $\mu_{1}$:Method Mk2 | -0.17986 | 0.04606 | -3.905 | < 0.001 |
| $\mu_{1}$:Method MP | -0.25017 | 0.04705 | -5.317 | < 0.001 |
| log(transitions):Method Mk2 | 0.17442 | 0.02398 | 7.275 | < 0.001 |
| log(transitions):Method MP | 0.13997 | 0.02405 | 5.819 | < 0.001 |


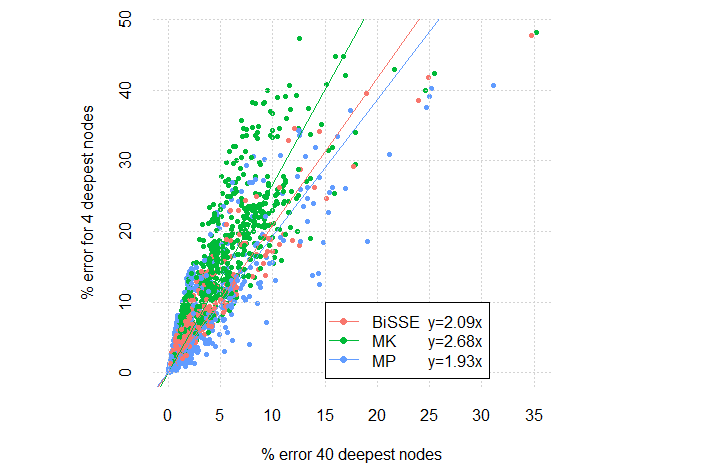


**Figure S1.** Mean error rates (based on raw scores) for the deepest decile compared to the deepest percentile for 720 different scenarios. The linear regressions reported in the legend are constrained to go through (0,0).


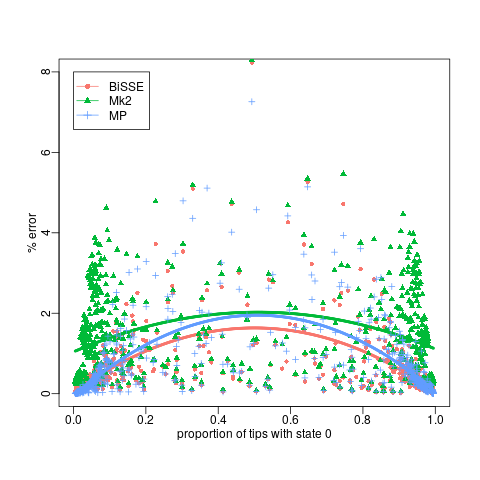


**Figure S2.** Error rates versus proportion of tips in state 0 for scenarios with both equal and unequal rates of speciation. Each point is the mean error rate for a parameter set for trees with 400 tips. The curves are quadratic regressions.


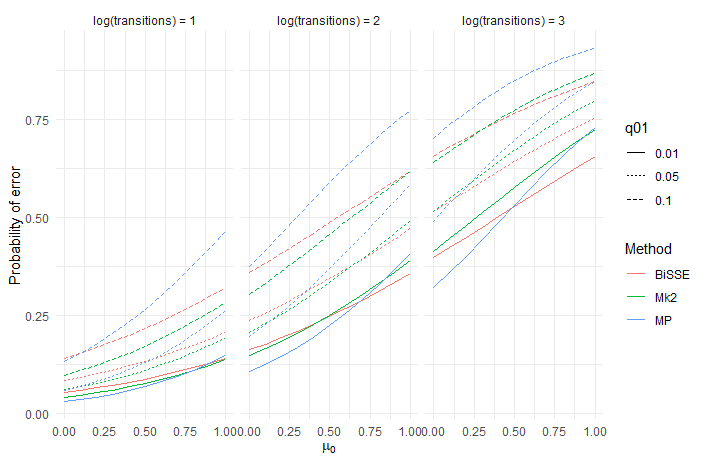


**Figure S3.** Predictions from a logistic regression model that fit the presence of an incorrect inferred state to the categorical main effect of method, the continuous main effects of log(#transitions), µ_0_, µ_1_, q_01_, q_10_ and interaction effects between Method and the other variables. The model was fit to data from 400 tip simulations across 500 repetitions of the 144 scenarios where λ_0_ = λ_1_= 1. For predictions $\mu_{1}$= 0.25 and q_10_ = 0.05. From left to right the panels show predictions where log(transitions) is equal to 1, 2 or 3.


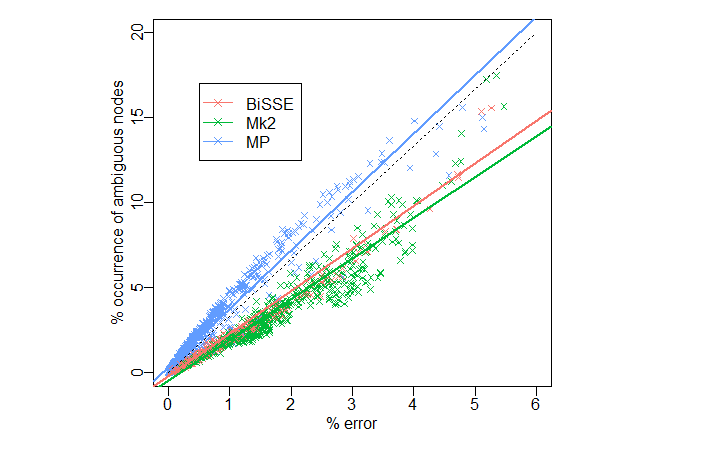


**Figure S****4.** Error rates (based on quantised scores) versus incidence of ambiguous nodes. Each point is the mean value for a parameter set for trees with 400 tips. The dashed line is the 1:1 relationships; solid lines are least squares regressions.


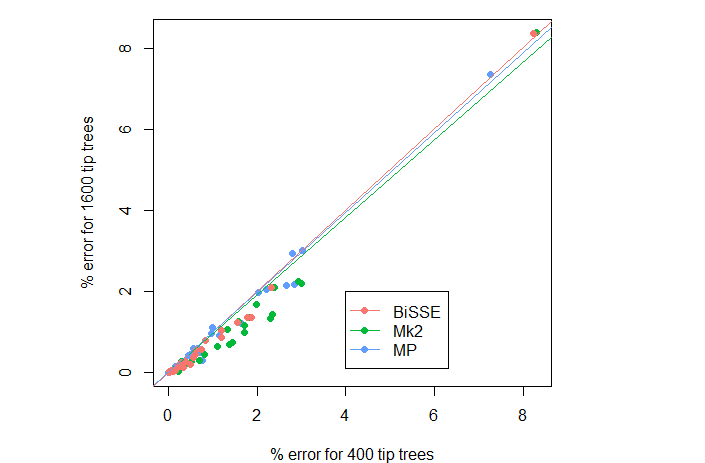


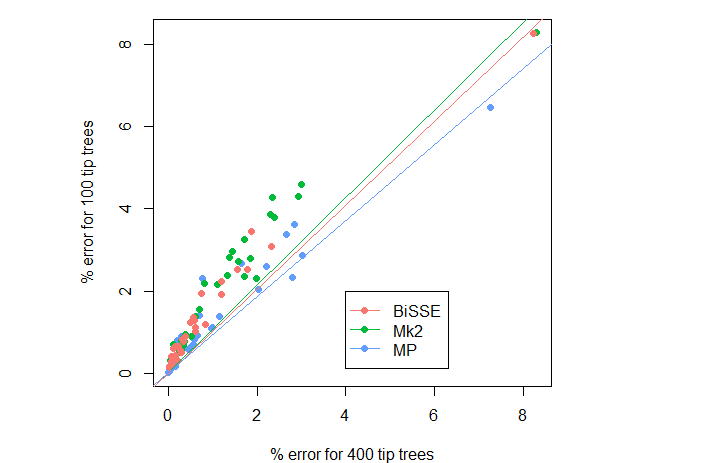


**Figure S5.** Comparison of error rates (based on raw scores) for 32 “corner case” scenarios with (A) 1600 tips or (B) 100 tips to error rates for scenarios with 400 tips.

a)

b)

**Figure S6**. Summary of a survey of 33 recent papers [references 1-33] using ancestral state reconstructions of discrete morphological/anatomical/behavioural characters (a total of 142 characters). These were the 33 most recent available papers (based on a search in Scopus for “ancestral” and “state” and “analysis”; access date 04/04/2019) that included reconstructions based on phylogenies with at least 10 tip taxa.

a) number of reconstructions binned by depth of the deepest inferred transition. 71% of reconstructions infer state changes in the deepest 20% of nodes in the tree.

b) number of reconstructions binned by number of inferred transitions measured as a percentage of the total number of edges (i.e. the number of places at which a transition could occur). 54% of reconstructions inferred transitions on at least 5% of the edges (equivalent to 40 transitions in a tree of 400 tips).

**References:**

[1] Borsch, T., Flores-Olvera, H., Zumaya, S. & Müller, K. 2018 Pollen characters and DNA sequence data converge on a monophyletic genus iresine (Amaranthaceae, caryophyllales) and help to elucidate its species diversity. *Taxon* **67**, 944-976. (doi:10.12705/675.7).

[2] Cairns, S.D. & Wirshing, H.H. 2018 A phylogenetic analysis of the Primnoidae (Anthozoa: Octocorallia: Calcaxonia) with analyses of character evolution and a key to the genera and subgenera. *BMC Evolutionary Biology* **18**. (doi:10.1186/s12862-018-1182-5).

[3] Castillo, E.R.D., Martí, D.A., Maronna, M.M., Scattolini, M.C., Cabral-de-Mello, D.C. & Cigliano, M.M. 2019 Chromosome evolution and phylogeny in Ronderosia (Orthoptera, Acrididae, Melanoplinae): clues of survivors to the challenge of sympatry? *Systematic Entomology* **44**, 61-74. (doi:10.1111/syen.12317).

[4] de Carvalho Dominguez Souza, B.F., König, A., Rasche, A., de Oliveira Carneiro, I., Stephan, N., Corman, V.M., Roppert, P.L., Goldmann, N., Kepper, R., Müller, S.F., et al. 2018 A novel hepatitis B virus species discovered in capuchin monkeys sheds new light on the evolution of primate hepadnaviruses. *Journal of Hepatology* **68**, 1114-1122. (doi:10.1016/j.jhep.2018.01.029).

[5] De-la-Mora, M., Piñero, D., Oyama, K., Farrell, B., Magallón, S. & Núñez-Farfán, J. 2018 Evolution of Trichobaris (Curculionidae) in relation to host plants: Geometric morphometrics, phylogeny and phylogeography. *Molecular Phylogenetics and Evolution* **124**, 37-49. (doi:10.1016/j.ympev.2018.02.018).

[6] Eldridge, M.D.B., Potter, S., Helgen, K.M., Sinaga, M.H., Aplin, K.P., Flannery, T.F. & Johnson, R.N. 2018 Phylogenetic analysis of the tree-kangaroos (Dendrolagus) reveals multiple divergent lineages within New Guinea. *Molecular Phylogenetics and Evolution* **127**, 589-599. (doi:10.1016/j.ympev.2018.05.030).

[7] Fernández-Mazuecos, M., Blanco-Pastor, J.L., Juan, A., Carnicero, P., Forrest, A., Alarcón, M., Vargas, P. & Glover, B.J. 2019 Macroevolutionary dynamics of nectar spurs, a key evolutionary innovation. *New Phytologist* **222**, 1123-1138. (doi:10.1111/nph.15654).

[8] Göpel, T. & Wirkner, C.S. 2018 Morphological description, character conceptualization and the reconstruction of ancestral states exemplified by the evolution of arthropod hearts. *PLoS ONE* **13**. (doi:10.1371/journal.pone.0201702).

[9] Hanschen, E.R., Herron, M.D., Wiens, J.J., Nozaki, H. & Michod, R.E. 2018 Multicellularity drives the evolution of sexual traits. *American Naturalist* **192**, E93-E105. (doi:10.1086/698301).

[10] Helm, C., Beckers, P., Bartolomaeus, T., Drukewitz, S.H., Kourtesis, I., Weigert, A., Purschke, G., Worsaae, K., Struck, T.H. & Bleidorn, C. 2018 Convergent evolution of the ladder-like ventral nerve cord in Annelida. *Frontiers in Zoology* **15**. (doi:10.1186/s12983-018-0280-y).

[11] Horká, I., De Grave, S., Fransen, C.H.J.M., Petrusek, A. & Ďuriš, Z. 2018 Multiple origins and strong phenotypic convergence in fish-cleaning palaemonid shrimp lineages. *Molecular Phylogenetics and Evolution* **124**, 71-81. (doi:10.1016/j.ympev.2018.02.006).

[12] Jeyarani, J.N., Yohannan, R., Vijayavalli, D., Dwivedi, M.D. & Pandey, A.K. 2018 Phylogenetic analysis and evolution of morphological characters in the genus Jasminum L. (Oleaceae) in India. *Journal of Genetics* **97**, 1225-1239. (doi:10.1007/s12041-018-1019-4).

[13] Jiang, F., Liu, Q., Liu, X., Wang, X.H. & Kang, L. 2019 Genomic data reveal high conservation but divergent evolutionary pattern of Polycomb/Trithorax group genes in arthropods. *Insect Science* **26**, 20-34. (doi:10.1111/1744-7917.12558).

[14] Kaveh, A., Kazempour-Osaloo, S., Amirahmadi, A., Maassoumi, A. & Schneeweiss, G.M. 2019 Systematics of Onobrychis sect. Heliobrychis (Fabaceae): morphology and molecular phylogeny revisited. *Plant Systematics and Evolution* **305**, 33-48. (doi:10.1007/s00606-018-1549-5).

[15] Kayal, E., Bentlage, B., Sabrina Pankey, M., Ohdera, A.H., Medina, M., Plachetzki, D.C., Collins, A.G. & Ryan, J.F. 2018 Phylogenomics provides a robust topology of the major cnidarian lineages and insights on the origins of key organismal traits. *BMC Evolutionary Biology* **18**. (doi:10.1186/s12862-018-1142-0).

[16] Kise, H., Maeda, T. & Reimer, J.D. 2019 A phylogeny and the evolution of epizoism within the family Hydrozoanthidae with description of a new genus and two new species. *Molecular Phylogenetics and Evolution* **130**, 304-314. (doi:10.1016/j.ympev.2018.10.011).

[17] Kobayashi, G., Goto, R., Takano, T. & Kojima, S. 2018 Molecular phylogeny of Maldanidae (Annelida): Multiple losses of tube-capping plates and evolutionary shifts in habitat depth. *Molecular Phylogenetics and Evolution* **127**, 332-344. (doi:10.1016/j.ympev.2018.04.036).

[18] Li, H., Sosa-Calvo, J., Horn, H.A., Pupo, M.T., Clardy, J., Rabeling, C., Schultz, T.R. & Currie, C.R. 2018 Convergent evolution of complex structures for ant-bacterial defensive symbiosis in fungus-farming ants. *Proceedings of the National Academy of Sciences of the United States of America* **115**, 10720-10725. (doi:10.1073/pnas.1809332115).

[19] Lusa, M.G., Loeuille, B.F.P. & Appezzato-da-Glória, B. 2018 First record of phytomelanin in aerial vegetative organs and its evolutionary implications in Lychnophorinae (Vernonieae: Asteraceae). *Perspectives in Plant Ecology, Evolution and Systematics* **33**, 18-33. (doi:10.1016/j.ppees.2018.04.006).

[20] Lusa, M.G., Loeuille, B.F.P., Ciccarelli, D. & Appezzato-da-Glória, B. 2018 Evolution of Stem and Leaf Structural Diversity: a Case Study in Lychnophorinae (Asteraceae). *Botanical Review* **84**, 203-241. (doi:10.1007/s12229-017-9191-4).

[21] Maciel, J.R., Louzada, R.B., Benko-Iseppon, A.M., Zizka, G. & Alves, M. 2018 Polyphyly and morphological convergence in Atlantic Forest species of Aechmea subgenus Chevaliera (Bromeliaceae). *Botanical Journal of the Linnean Society* **188**, 281-295. (doi:10.1093/botlinnean/boy053).

[22] Monson, T.A. & Hlusko, L.J. 2018 Breaking the rules: Phylogeny, not life history, explains dental eruption sequence in primates. *American Journal of Physical Anthropology* **167**, 217-233. (doi:10.1002/ajpa.23618).

[23] Nagel, J.H., Wingfield, M.J. & Slippers, B. 2018 Evolution of the mating types and mating strategies in prominent genera in the Botryosphaeriaceae. *Fungal Genetics and Biology* **114**, 24-33. (doi:10.1016/j.fgb.2018.03.003).

[24] Ortiz, D., Francke, O.F. & Bond, J.E. 2018 A tangle of forms and phylogeny: Extensive morphological homoplasy and molecular clock heterogeneity in Bonnetina and related tarantulas. *Molecular Phylogenetics and Evolution* **127**, 55-73. (doi:10.1016/j.ympev.2018.05.013).

[25] Pérez-González, S., Andújar, C. & Zaballos, J.P. 2018 Hidden biodiversity: total evidence phylogenetics and evolution of morphological traits in a highly diverse lineage of endogean ground beetles, Typhlocharis Dieck, 1869 (Carabidae, Trechinae, Anillini). *Cladistics* **34**, 359-383. (doi:10.1111/cla.12208).

[26] Schafran, P.W., Zimmer, E.A., Taylor, W.C. & Musselman, L.J. 2018 A Whole Chloroplast Genome Phylogeny of Diploid Species of Isoëtes (Isoëtaceae, Lycopodiophyta) in the Southeastern United States. *Castanea* **83**, 224-235. (doi:10.2179/17-132).

[27] Silva-Luz, C.L.D., Pirani, J.R., Mitchell, J.D., Daly, D., Capelli, N.D.V., Demarco, D., Pell, S.K. & Plunkett, G.M. 2019 Phylogeny of Schinus L. (Anacardiaceae) with a new infrageneric classification and insights into evolution of spinescence and floral traits. *Molecular Phylogenetics and Evolution* **133**, 302-351. (doi:10.1016/j.ympev.2018.10.013).

[28] Soliz, M.C., Ponssa, M.L. & Abdala, V. 2018 Comparative anatomy and development of pectoral and pelvic girdles in hylid anurans. *Journal of Morphology* **279**, 904-924. (doi:10.1002/jmor.20820).

[29] Sosa-Calvo, J., Fernández, F. & Schultz, T.R. 2019 Phylogeny and evolution of the cryptic fungus-farming ant genus Myrmicocrypta F. Smith (Hymenoptera: Formicidae) inferred from multilocus data. *Systematic Entomology* **44**, 139-162. (doi:10.1111/syen.12313).

[30] Testo, W., Øllgaard, B., Field, A., Almeida, T., Kessler, M. & Barrington, D. 2018 Phylogenetic systematics, morphological evolution, and natural groups in neotropical *Phlegmariurus* (Lycopodiaceae). *Molecular Phylogenetics and Evolution* **125**, 1-13. (doi:10.1016/j.ympev.2018.03.016).

[31] Tseng, Y.H., Monro, A.K., Wei, Y.G. & Hu, J.M. 2019 Molecular phylogeny and morphology of Elatostema s.l. (Urticaceae): Implications for inter- and infrageneric classifications. *Molecular Phylogenetics and Evolution* **132**, 251-264. (doi:10.1016/j.ympev.2018.11.016).

[32] Yang, F., Chang, W., Hayashi, F., Gillung, J., Jiang, Y., Yang, D. & Liu, X. 2018 Evolutionary history of the complex polymorphic dobsonfly genus Neoneuromus (Megaloptera: Corydalidae). *Systematic Entomology* **43**, 568-595. (doi:10.1111/syen.12287).

[33] Zattara, E.E., Fernández-Álvarez, F.A., Hiebert, T.C., Bely, A.E. & Norenburg, J.L. 2019 A phylum-wide survey reveals multiple independent gains of head regeneration in Nemertea. *Proceedings. Biological \Sciences* **286**, 20182524. (doi:10.1098/rspb.2018.2524).
